# Utilization of a Wheat55K SNP Array for Mapping of Major QTL for Temporal Expression of the Tiller Number

**DOI:** 10.3389/fpls.2018.00333

**Published:** 2018-03-15

**Authors:** Tianheng Ren, Yangshan Hu, Yingzi Tang, Chunsheng Li, Benju Yan, Zhenglong Ren, Feiquan Tan, Zongxiang Tang, Shulan Fu, Zhi Li

**Affiliations:** ^1^College of Agronomy, Sichuan Agricultural University, Chengdu, China; ^2^College of Life Science, Sichuan Agricultural University, Ya’an, China

**Keywords:** *Triticum aestivum* L., Wheat55K SNP Array, tiller number, QTL, genetic mapping

## Abstract

Maximum tiller number and productive tiller number are important traits for wheat grain yield, but research involving the temporal expression of tiller number at different quantitative trait loci (QTL) levels is limited. In the present study, a population set of 371 recombined inbred lines derived from a cross between Chuan-Nong18 and T1208 was used to construct a high-density genetic map using a Wheat55K SNP Array and to perform dynamic QTL analysis of the tiller number at four growth stages. A high-density genetic map containing 11,583 SNP markers and 59 SSR markers that spanned 4,513.95 cM and was distributed across 21 wheat chromosomes was constructed. A total of 28 single environmental QTL were identified in the recombined inbred lines population, and among these, seven QTL were stable and used for multi-environmental and dynamic analysis. These QTL were mapped to chromosomes 2D, 4A, 4D, 5A, 5D, and 7D, respectively. Each QTL explained 1.63–21.22% of the observed phenotypic variation, with an additive effect from -20.51 to 11.59. Dynamic analysis showed that *cqTN-2D.2* can be detected at four growth stages of tillering, explaining 4.92–17.16% of the observed phenotypic variations and spanning 13.71 Mb (*AX-109283238-AX-110544009*: 82189047-95895626) according to the physical location of the flanking markers. The effects of the stable QTL were validated in the recombined inbred lines population, and the beneficial alleles could be utilized in future marker-assisted selection. Several candidate genes for MTN and PTN were predicted. The results provide a better understanding of the QTL selectively expressing the control of tiller number and will facilitate future map-based cloning. 9.17% SNP markers showed best hits to the Chinese Spring contigs. It was indicated that Wheat55K Array was efficient and valid to construct a high-density wheat genetic map.

## Introduction

Common wheat (*Triticum aestivum* L.) is one of the most valuable and widely used crops in the world. The grain yield of wheat was 28,462.68 bushels for food use in 2016 according to the United States Department of Agriculture^1^. In response to the continuous decrease in wheat-cultivated areas and growth in population size, the major object of recent wheat breeding programs is to improve grain yield. Grain yield in wheat is a complex trait that is usually controlled by several quantitative trait loci (QTL) and is also significantly influenced by numerous environment factors. Tiller number (TN) is an important agronomic trait and plays a large role in wheat for yield potential because maximum tiller number per unit area (MTN) determines panicle number per unit area, also known as productive tiller number (PTN), which is a key component of grain yield (Naruoka et al., 2011). In addition, tillering determines shoot architecture, which affects the synchrony of flowering, illumination, plant height, seed set, and ultimately, the grain yield of each plant (Kuraparthy et al., 2007; Xu et al., 2017). However, TN is a trait for which gene expression is dynamical and can easily be measured (Liu et al., 2009). Thus, TN is a model trait for studying developmental behavior (Yan et al., 1998). Previous studies showed that wheat cultivars with limited tillering capacity have higher yield potential than cultivars with more tillers under severe drought conditions (Richards, 1988; Naruoka et al., 2011; Wang et al., 2016). However, Sichuan Province is a typical rain-fed agricultural area in Southwestern China that has very few light hours and more rainy days in winter. This has resulted in a macro-type spike and low tillering-capacity winter wheat with high-density sowing in recent years, but this may change. Macro-type spikes and limited tillering capacity can cause poor resistance to extreme weather, and the main culm may be injured by extreme weather such as frost, which can devastatingly reduce grain yield (Fowler, 1982). More seriously, under high-density sowing conditions, the appearance of flag leaves increases the number of leaves and leaf area, thereby increasing the shadow effect. A larger shadow effect and higher soil humidity can give rise to widespread disease (Kumar and Nagarajan, 1998). Furthermore, there is a highly negative correlation between TN and plant height (Lanning et al., 2012; Lu et al., 2015; Xu et al., 2017). Thus, new breeding hypotheses for wheat in Sichuan Basin and similar environments is to increase TN appropriately to construct an ideal population structure. According to this thinking, our breeding produced wheat cultivars such as CN12, CN17, and CN18 with higher tillering capacity. Previous studies have paid more attention to the cultivation methods affecting TN, such as seeding density and sowing time. However, our understanding of the genetic basis of TN is limited. Thus, to increase TN in future breeding programs, it is essential to elucidate the genetic mechanism controlling TN in wheat.

Quantitative trait loci mapping that utilizes molecular markers is an effective approach to dissect complex traits in crops (Gao et al., 2015; Zhai et al., 2016; Yuan et al., 2017). Studies using molecular markers began with restriction fragment length polymorphisms (RFLPs) three decades ago and have recently culminated with single nucleotide polymorphism (SNP) (Tanksley et al., 1989; Rasheed et al., 2017). SNPs are the most abundant type of molecular markers, and the development and application of SNP arrays based on next-generation sequencing technologies in crop genetics have gained remarkable attention in recent years (Varshney et al., 2009; Rasheed et al., 2017). Several SNP arrays of wheat have been reported, such as 9K, 90 K, 35K, 660K, and 820K (Cavanagh et al., 2013; Wang et al., 2014; Winfield et al., 2016; Allen et al., 2017; Cui et al., 2017). Recently, a high-density genetic map has been used for several traits in wheat (Wu et al., 2015; Cui et al., 2017; Yu et al., 2017), providing useful markers and information for genetic analysis and breeding. The population size is also important for QTL mapping because it affects accuracy. Limited populations can cause overestimation of QTL and can explain phenotypic variations and detection of false positive QTL (Vales et al., 2005; Cui et al., 2011). Previous research has shown that a threshold of population size for QTL mapping is 300 (Li et al., 2006). Using traditional genetic analysis, there have been several reports of QTL controlling TN in wheat. To date, at least four single genes (*tin1*, *tin2*, *tin3*, and *ftin*) that control TN have been reported (Peng et al., 1998; Spielmeyer and Richards, 2004; Kuraparthy et al., 2007; Zhang et al., 2013). To date, several QTL have been associated with TN mapped to chromosomes 1A, 2B, 2A, 2B, 2D, 3A, 3B, 3D, 4D, 5A, 5D, 6D, 7A, and 7B (Kato et al., 2000; Li et al., 2010; Deng et al., 2011; Naruoka et al., 2011; Nasseer et al., 2016; Wang et al., 2016; Hu et al., 2017; Xu et al., 2017). According to the theory of developmental genetics, genes and QTL are expressed temporally at different growth stages (Zhu, 1995), even though they may have the same ultimate effects. The dissection of tillering dynamics into component traits can provide insight into the interaction of phenotypes and environments in crops (Hammer et al., 2006). The observation of phenotypic expression of component traits is generally more stable across environments than the expression of the complex trait itself, indicating that component traits facilitate identification of QTL or genes (Alam et al., 2014). There are several dynamic QTL analyses for TN in rice (*Oryza sativa* L.) (Liu et al., 2009, 2012), and despite the importance of tillering dynamics in potential grain yield, the understanding and investigation of the genetic basis and environmental factors affecting TN in wheat are limited.

The Wheat660K and Wheat55K Array were both designed by the Chinese Academy of Agricultural Sciences and synthesized by Affymetrix. Wheat660K is efficient and valid to construct a high-density wheat genetic map (Cui et al., 2017). Compared to Wheat660K, Wheat55K (Affymetrix^®^ Axiom^®^ Wheat55) is more valuable for wheat QTL research, because all 53063 tags of the 55K Array were carefully selected from the 660K Array. These tags were uniformly distributed in 21 wheat chromosomes, and each chromosome had approximately 2600 SNP. The average genetic distance is approximately 0.1 cM, and the average physical distance was smaller than 300 kb.

In the present study, a set of 371 recombined inbred lines (RILs) were used to construct a high-density linkage map by using the Wheat55K SNP Array and to perform dynamic QTL analysis for TN at four growth stages. This is the first report of the use of 55K SNP Array in wheat genetic mapping. The sequences of the flanking SNP marker were employed in BLASTN against Chinese Spring contigs, and the beneficial alleles were identified in the RIL population. These QTL detected in the RIL population will be useful for the development of grain yield and further marker-assisted selection (MAS) and map-based cloning.

## Materials and Methods

### Plant Materials and Phenotypic Evaluation

Chuan-Nong18 (CN18) was examined and approved as a cultivar by the Sichuan Provincial Variety Examination and Committee in 2003, and the high-tillering and high-yielding characters of CN18 were found to be stably inherited (Ren et al., 2011). T1208 was developed by our lab and exhibited significant differences in tillering numbers compared to CN18. In the present study, 371 F_11:12_ RILs for integrated linkage map and QTL analyses were developed from a cross between CN18 and T1208 by single-seed descent. The parental lines and 371 RILs were planted in the Qionglai District (30°25′N, 103°28′, altitude 493.3 m) in Sichuan Basin during the 2014.10–2015.05 cropping season and 2015.10–2016.05 cropping season following standard cultivation practices. A randomized block design with three replicates was used for this population. Forty seeds were hand-planted in each row of a four-row plot with a row spacing of 25 cm and length of 2 m. Crop management was performed according to local practices. Fungicide was applied to the seedlings and against at heading to control diseases and pests.

Twenty representative plants from the middle of inner rows of each replicate were selected to measure the TN across four different growth stages: 30 days after seeding, 49 days after seeding, 79 days after seeding, and 157 days after seeding (Zadoks et al., 1974). The trait names were designated tiller number at early stage per unit area (TNES), tiller number during pre-winter per unit area (TNPW), maximum tiller number per unit area (MTN), and productive tiller number per unit area (PTN), respectively. Per unit area for trait measurement was 1 m^2^ in the present study. The particular phenotyping evaluation was as described in Hu et al. (2017).

### Molecular Genotyping and Map Construction

Genomic DNA for the 371 RILs and their parental lines was extracted from tender leaves using the Trelief^TM^ Plant Genomic DNA Kit^2^, and quality was checked using an agarose gel. The concentration was detected using an Thermo Scientific NanoDrop 2000. The qualified DNA was genotyped using the Affymetrix 55K SNP Array by CapitalBio Technology Company (Beijing, China). Chip genotyping was according to the Axiom^®^ 2.0 Assay for 384 Samples User Manual^3^. The SSR markers were genotyped in our previous study (Hu et al., 2017).

Biallelic pleomorphic SNPs with >10% missing data and *p* < 10^-6^ by chi-square test of segregation distortion (departure from the expect 1:1 segregation ratio) were removed, and the remaining high-quality SNPs were binned by their pattern of segregation using the BIN function of IciMapping Ver. 4.1^4^ (Winfield et al., 2016; Zhang et al., 2017). Each bin had several markers; the correlation coefficient between them was 1, and one marker with the lowest missing rate was chosen to represent this bin. If there was no missing date of the markers in one bin, one marker was chosen randomly. SNP markers and the previously mapped 59 SSR markers were sorted into groups using Carthagene (Givry et al., 2005). LOD thresholds between 5.0 and 10.0 and a threshold of genetic distance of 80 cM were tested to reach an optimum marker number in linkage groups. The linkage map was constructed using the MAP function of IciMapping Ver. 4.1, and the algorithm of groups ordered was “nnTwoOpt” using the Kosambi map function to calculate the map distance from recombination frequencies (Kosambi, 1943). The criterion and window size for Rippling were “SARF” and “5,” respectively. MapChart Ver. 2.2^5^ was used for genetic map drawing. The SNP markers mapped to the genetic map were employed to perform BLASTX search on ENSEMBL^6^.

### Statistical and QTL Analysis

The average measurement of TNES, TNPW, MTN, and PTN of three replicates was used in the subsequent analysis. The heritability and ANOVA of each trait were performed as described (Hu et al., 2017). The correlation was performed using SPSS Ver. 22.0 (IBM SPSS 22.0, Chicago, IL, United States).

The QTL for TN at different growth stages were identified using the IciMapping Ver. 4.1 BIP functionality with the inclusive composite interval mapping (ICIM) method, and each trait value in each replicate of the 2 years was assembled to conduct combined QTL analysis to identify the cQTL with additive-by-environment (A by E) interaction effects in a multi-environment trials (MET) module using the ICIM method. For each trial, a test of 2,000 permutations was performed to identify the LOD threshold corresponding to a genome-wild false discovery rate of 0.01. The walking step for QTL mapping was 0.1 cM with a *p*-value inclusion threshold (probability in stepwise regression) of 0.001 (Li et al., 2007; Meng et al., 2015). The confidence interval of each QTL was determined by LOD peak -2. A QTL detected in both years at the same growth stage or at least three growth stages was considered a stable QTL. The naming rule of each QTL was designated *q* and *cq* for QTL and combined QTL, with *TN* (including TNES, TNPW, MTN, PTN) for trait name, along with the chromosome information at the end. The sequences of the flanking markers of the stable QTL were employed to perform BLASTN against the Chinese Spring contigs^7^. The overlapping confidence intervals and the flanking sequences of SNP markers were used to predict the candidate genes by IWGSC WGA of chromosome 2D and 4D^8^.

## Results

### Phenotyping Evaluation and Correlation Between TNES, TNPW, MTN, and PTN

Our previous research has shown that the above four traits followed a normal distribution in the RIL population, and the phenotype of two parental lines showed a great discrepancy, indicating that this RIL population is suitable for QTL analysis (Hu et al., 2017). In results using the average measurement of TNES, TNPW, MTN, and PTN between 2 years for correlation analysis, traits were significantly positive correlated with each other in the RIL population (**Table 1**). The highest correlation coefficient was 0.792 (*p* < 0.001) between MTN and PTN (**Table 1**). The mean values of TNES, TNPW, MTN, and PTN in the RIL population for the 2 years are shown in **Supplementary Figure S1**. These four traits showed an obviously normal distribution in the population.

**Table 1:** Correlation coefficient among various tiller number-related traits in RIL population.

Traits	TNES	TNPW	MTN	PTN
TNES	1			
TNPW	0.738^∗∗∗^	1		
MTN	0.415^∗∗∗^	0.663^∗∗∗^	1	
PTN	0.395^∗∗∗^	0.588^∗∗∗^	0.792^∗∗∗^	1


*^∗∗∗^Indicates extremely significant correlation at 0.001 probability level. TNES, tiller number at early stage per unit area; TNPW, tiller number during pre-winter per unit area; MTN, maximum tiller number per unit area; PTN, productive tiller number per unit area.*

### Genetic Map Construction

An RIL population of 371 lines derived from CN18 and T1208 was used to construct the genetic map. Out of 55K SNP markers, approximately 17K (∼30%) were polymorphic between the parental lines. After removing SNPs with more than 10% missing data or a segregation distortion test *p* < 10^-6^, a novel high-density genetic map consisting of 11,642 markers spanning 4,513.95 cM and distributed across 21 wheat chromosomes was constructed (**Table 2** and **Supplementary Figure S2**). For the map length, the A, B, and D genomes spanned 1,169.38 cM (25.91%), 1,394.97 cM (30.90%), and 1949.60 cM (43.19%), respectively. The genetic length of different chromosomes varied from 120.52 cM (1A) to 320.21 cM (2D), averaging 214.95 cM. Among these markers, 11,583 were SNPs derived from the Affymetrix Wheat55K SNP Array, and the remaining 59 markers were SSR markers. These 11,642 markers had 2,001 patterns of segregation in the RIL population, and hence, 2,001 markers with the lowest missing rate in each bin were chosen to represent the corresponding bin and are shown in the genetic map. The number of markers in each bin ranged from 1 to 989 (detailed marker information for each bin is shown in **Supplementary Table S1**). The bin number of different chromosomes ranged from 40 to 164, and the average loci density and marker density of the genetic map were 2.26 cM and 0.39 cM, respectively. Only 1 gap between 30 cM and 40 cM on chromosome 3D was found in the genetic map. In order to specify the coding region SNP markers mapped to the CN18/T1208 RIL population, the BLASTX search was performed by using the flanking sequences of SNP markers as queries on ENSEMBL. Out of the 11,583 SNP markers, 1,062 SNP (9.17%) markers showed best hits to the Chinese Spring contigs (**Supplementary Table S3**). Thus, these SNP markers were considered to be the coding region SNP.

**Table 2:** Distribution of mapped markers on 21 chromosomes in genetic map constructed by CN18/T1208 RIL population.

Chromosomes	Length (cM)	Marker number^a^	Bin number^b^	Resolution (cM)^c^	Average (cM)^d^
1A	120.52	388	62	0.31	1.94
1BL	182.22	173	59	1.05	3.09
1D	306.86	701	93	0.44	3.30
2A	157.53	1,194	75	0.13	2.10
2B	265.34	764	142	0.35	1.87
2D	320.21	764	115	0.42	2.78
3A	198.52	316	83	0.63	2.39
3B	212.76	680	83	0.31	2.56
3D	270.95	327	83	0.83	3.26
4A	169.91	374	90	0.45	1.89
4B	175.59	1,038	101	0.17	1.74
4D	295.34	292	93	1.01	3.18
5A	198.34	366	84	0.54	2.36
5B	217.76	974	164	0.22	1.33
5D	257.51	585	131	0.44	1.97
6A	114.32	150	40	0.76	2.86
6B	159.78	493	108	0.32	1.48
6D	287.67	433	98	0.66	2.94
7A	210.24	508	104	0.41	2.02
7B	181.52	583	87	0.31	2.09
7D	211.06	539	106	0.39	1.99
Total	4,513.95	11,642	2,001	0.39	2.26


*^*a*^Distribution of 11,642 markers on the 21 wheat chromosomes. SNP markers were derived from Affymetrix 55K SNP Array, and SSR markers were obtained from the United States Department of Agriculture (https://wheat.pw.usda.gov). ^*b*^The 11,642 markers had 2,001 patterns of segregation; hence, 2,001 markers were chosen to represent each bin in genetic map, and bins were defined as loci on each chromosome and consisted of at least 1 marker. ^*c*^Resolution means the average distance between two linked markers. ^*d*^Average means the average distance between two neighbor loci (bins) according to the genetic map.*

### QTL Analysis in Individual Environments

**FIGURE 1:**
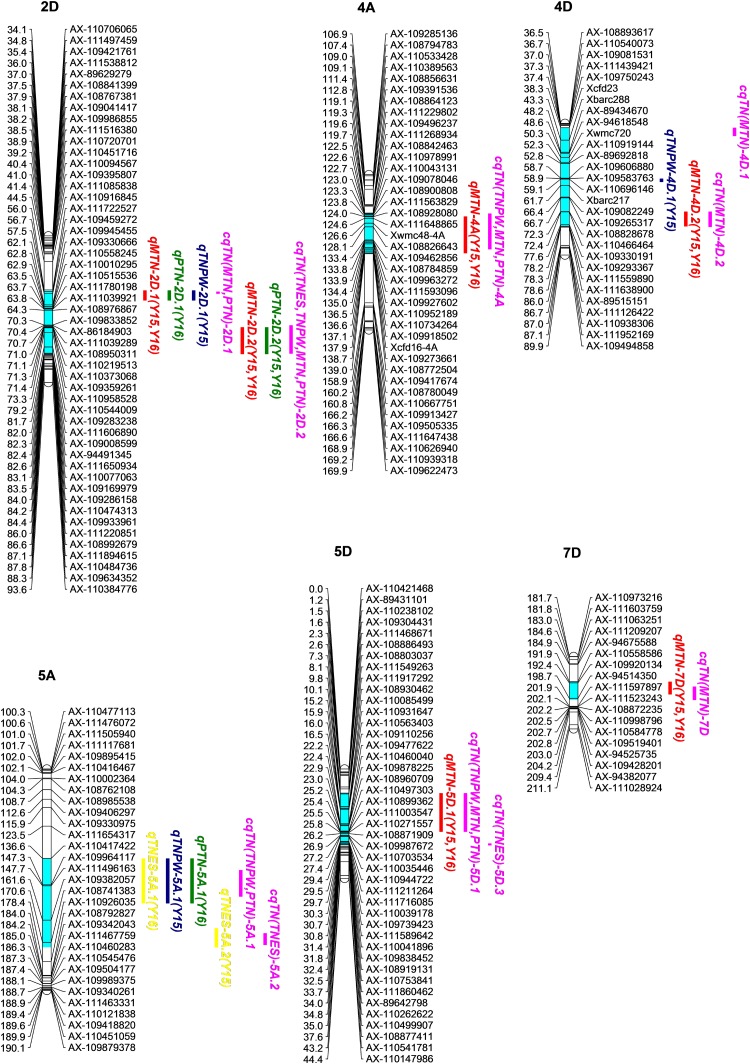
Chromosomal locations of the seven stable QTL associated with TNES, TNPW, MTN, and PTN based on a 371 RIL population derived from a across between CN18 and T1208. QTL intervals were determined by LOD-2 from the peak. The putative QTL clusters were marked clearly on the chromosomes.

Using the ICIM method, a total of 28 additive QTL for TNES, TNPW, MTN, and PTN were identified within at least 1 year (**Table 3**). The LOD thresholds for each trait were 4.06, 4.22, 4.23, and 4.08, respectively. If the confidence interval of the corresponding QTL were overlapped, these QTL were considered, and named, as one QTL. The putative additive QTL were mapped to chromosomes 1A, 2B, 2D, 4A, 4D, 5A, 5D, 6D, and 7D, respectively (**Figure 1** and **Table 3**). The LOD values of each QTL ranged from 4.88 to 35.11, with an additive effect from -37.66 to 18.71, and each QTL explained 0.74–19.39% of the observed phenotypic variation. At the MTN and PTN stages, *qTN-2D.1*, *qTN-2D.2*, *qTN-4A*, *qTN-4D.2*, *qTN-5D.1*, and *qTN-7D* were identified in both years. *qTN-2D.1*, *qTN-2D.2*, and *qTN-5A.1* were identified in at least three growth stages. The QTL mentioned above were considered stable QTL. The seven stable QTL were verified in combined QTL analysis. For *qTN-2D.1*, *qTN-2D.2*, *qTN-5A.1*, *qTN-5D.1*, and *qTN-7D*, the positive effects that increased TN were derived from CN18. Conversely, the positive effects were provided by T1208 for the remaining two QTL.

**Table 3:** Putative additive QTL for TN at different growth stages by ICIM method.

QTL^a^	Interval (cM)	Stages^b^	Year	LOD	PVE (%)^c^	Add^d^
***qTN-1A***	48.15–65.05	TNES	Y16	15.27	3.97	4.06
*qTN-2B*	98.05–108.85	TNPW	Y16	7.00	9.44	13.00
		TNES	Y16	8.63	1.12	9.16
***qTN-2D.1***	56.25–60.15	TNPW	Y15	5.58	6.60	-5.97
		MTN	Y15/Y16	10.73/18.67	5.07/18.67	-19.12/-26.28
		PTN	Y16	10.14	10.39	-8.73
***qTN-2D.2***	71.25–81.95	MTN	Y15/Y16	35.11/4.45	19.34/3.85	-37.66/-13.18
		PTN	Y15/Y16	11.14/17.84	9.91/19.39	-12.02/-13.33
***qTN.4A***	123.95–138.65	MTN	Y15/Y16	10.36/6.72	5.79/2.80	18.71/15.51
*qTN-4D.1*	58.95–60.15	TNPW	Y15	10.95	13.36	8.45
***qTN-4D.2***	72.35–78.15	MTN	Y15/Y16	21.17/15.50	11.00/14.04	28.10/23.58
***qTN-5A.1***	136.65–154.65	TNES	Y16	6.99	0.74	-6.02
		TNPW	Y15	9.56	12.88	-8.38
		PTN	Y16	8.13	9.01	-8.12
*qTN-5A.2*	164.95–172.55	TNES	Y15	10.51	9.83	-4.12
***qTN-5D.1***	9.85–25.35	MTN	Y15/Y16	6.68/4.61	3.09/3.92	-15.61/-12.79
*qTN-5D.2*	211.35–225.15	MTN	Y15	6.73	3.09	-15.26
		PTN	Y16	6.17	6.14	6.69
*qTN-6D*	276.26–280.66	MTN	Y15	4.88	2.22	13.22
		PTN	Y15	5.26	4.79	9.76
***qTN-7D***	191.95–196.95	MTN	Y15/Y16	5.78/4.80	2.68/4.64	-14.29/-14.01


*^*a*^Stable QTL are marked in ***bold*** typeface. A QTL detected in 2 years or at least three individual growth stages was considered stable. ^*b*^TNES, TNPW, MTN, and PTN represent the four growth stages of tillering. ^*c*^Proportion of phenotypic variation of the corresponding QTL. ^*d*^Positive additive effects indicate that alleles from T1208 enhance corresponding trait values, and negative additive effects indicate that alleles from CN18 enhance corresponding trait values.*

### Combined QTL by Environment Interaction Analysis

The results of using the MET module for combined QTL by environment interaction analysis of the seven putative stable QTL showed that all the QTL were detected across the different tiller growth stages (**Figure 1** and **Table 4**). The LOD values of the cQTL ranged from 4.47 to 69.52, and each cQTL explained 1.63–21.22% of the observed phenotypic variation. The LOD (A) and LOD (AE) of the seven stable cQTL were 1.55–39.73 and 1.35–54.70, respectively. Among these cQTL, *cqTN-2D.2* could be detected in all tiller growth stages, two cQTL (*cqTN-4A* and *cqTN-5D.1*) could be detected in three growth stages, and two cQTL (*cqTN-2D.1* and *cqTN-5A.1*) could be detected in two growth stages. The additive effects of *cqTN-2D.1*, *cqTN-2D.2*, *cqTN-5A.1*, *cqTN-5D.1*, and *cqTN-7D* were negative, indicating that alleles from CN18 enhance TN across different growth stages at these QTL levels. The chromosomal location of all QTL showed six QTL clusters mapped to 2D, 4A, 4D, 5A, 5D, and 7D, respectively (**Figure 1** and **Table 5**). These QTL clusters contained 14 single environmental QTL and 19 combined QTL associated with TN. QTL clusters C1, C3, C4, and C5 contained major QTL. In C1, C4, C5, and C6, beneficial alleles derived from CN18 contributed to increased TN at different growth stages. In C2, alleles from T1208 increased TN. C1 is a 25.70 cM region on chromosome 2D that consists of 11 QTL associated with all traits (**Table 5**).

**Table 4:** Combined QTL analysis for stable QTL.

cQTL^a^	Interval (cM)	Stage	LOD	LOD (A)^b^	LOD (AE)^c^	%PVE	%PVE (A)^d^	%PVE (AE)^e^	Add^f^
*cqTN-2D.1*	56.75–57.45	MTN	37.27	32.00	5.27	10.99	8.45	2.54	-19.45
		PTN	22.60	9.66	12.94	6.01	3.23	2.78	-6.20
*cqTN-2D.2*	70.45–81.75	TNES	4.47	1.80	2.67	4.92	2.43	2.49	-3.69
		TNPW	8.19	4.82	3.37	6.90	3.06	3.84	-4.50
		MTN	55.28	35.39	19.89	13.62	9.22	4.40	-20.51
		PTN	47.05	39.73	7.32	17.16	13.25	3.91	-12.69
*cqTN-4A*	122.75–137.05	TNPW	5.34	1.55	3.79	1.73	1.00	0.73	2.53
		MTN	11.42	4.68	6.74	2.88	1.20	1.68	7.23
		PTN	7.48	6.13	1.35	2.64	2.00	0.64	4.89
*cqTN-4D.2*	72.35–78.35	MTN	25.67	11.86	13.81	6.48	3.07	3.41	11.59
*cqTN-5A.1*	141.35–151.95	TNPW	10.64	5.07	5.57	3.70	3.19	0.51	-4.56
		PTN	9.00	5.30	3.70	2.36	1.65	0.71	-4.43
*cqTN-5D.1*	9.85–25.35	TNPW	5.59	2.66	2.93	3.55	1.72	1.83	-3.76
		MTN	69.52	14.82	54.70	21.22	3.87	17.35	-13.43
		PTN	7.26	3.17	4.09	1.63	0.99	0.64	-3.78
*cqTN-7D*	193.95–199.25	MTN	10.02	7.12	2.90	2.33	1.70	0.63	-8.98


*^*a*^cQTL represents QTL identified by combined QTL analysis of multi-environment trials. ^*b,c*^LOD (A) represents LOD score for additive and dominance effects. LOD (AE) represents LOD score for additive and dominance by environment effects. ^*d,e*^%PVE (A) and %PVE (AE) represent phenotypic variation explained by additive and dominance effects and additive and dominance by environment effects for corresponding QTL, respectively.*

**Table 5:** QTL clusters simultaneously affecting TNES, TNPW, MTN, and PTN in CN18/T1208 RIL population.

Cluster	Chr.	Interval (cM)	No. of QTL	QTL (additive effects)^a^
C1	2D	56.25–81.95	11	*qTNPW-2D.1* (-5.97), ***qMTN-2D.1* (**-**22.70)**, ***qPTN-2D.1* (**-**8.73)**, ***qMTN-2D.2* (**-**25.42)**, ***qPTN-2D.2* (**-**12.68)**, ***cqMTN-2D.1* (**-**19.45)**, *cqPTN-2D.1* (-6.20), *cqTNES-2D.2* (-3.69), *cqTNPW-2D.2* (-4.50), ***cqMTN-2D.2* (**-**20.51)**, *cqPTN-2D.2* (-12.69)
C2	4A	122.75–138.65	4	*qMTN-4A* (17.11), *cqTNPW-4A* (2.53), *cqMTN-4A* (-7.23), *cqPTN-4A* (4.89)
C3	4D	38.32–78.35	5	***qTNPW-4D.1* (8.45)**, ***qMTN-4D.2* (25.84)**, *cqTNPW-4D.1* (2.44), *cqTNES-4D.1* (1.76), *cqMTN-4D.2* (11.59)
C4	5A	136.65–172.55	7	*qTNES-5A.1* (-3.01), ***qTNPW-5A.1* (**-**8.38)**, *qPTN-5A.1* (-8.12), *qTNES-5A.2* (-4.12), *cqTNPW-5A.1* (-4.56), *cqPTN-5A.1* (-4.43), *cqTNES-5A.2* (-2.91)
C5	5D	9.85–30.85	4	*qMTN-5D.1* (-14.2), *cqTNPW-5D.1* (-3.76), ***cqMTN-5D.1* (**-**13.43)**, *cqPTN-5D.1* (-3.78)
C6	7D	191.95–199.25	2	*qMTN-7D* (-14.15), *cqMTN-7D* (-8.98)


*^*a*^Major QTL are in ***bold*** typeface. QTL with LOD > 3 and PVE > 10% was considered major QTL.*

The flanking sequences of the closet SNP markers of the seven stable QTL were used in BLASTN against the Chinese Spring contigs. *cqTN-2D.1*, *cqTN-2D.2*, *cqTN-4A*, *cqTN-4D.2*, *cqTN-5A.1*, and *cqTN-7D* showed the best hits to chromosomes 2DS, 2DS, 4AL, 4DL, 5AL, and 7DS, respectively. In addition, *cqTN-5D.1* showed the best hits for both 5DS and 5DL. All flanking SNP markers showed 100% hits against the chosen Chinese Spring contigs. By comparing the flanking markers to the physical locations, these QTL spanned 11.68 Mb, 13.71 Mb, 21.98 Mb, 7.36 Mb, 62.46 Mb, 40.50 Mb, and 26.98 Mb, respectively. The candidate genes prediction of *cqTN-2D.2* (*AX-109283238-AX-110544009*: 82189047-95895626) and *cqTN-4D.2* (*AX-109330191-AX-110466464*: 374963016-382320415) was performed by the flanking sequences of linkage SNP markers. The results showed that 136 and 56 candidate genes in the mentioned above regions of chromosome 2D and chromosome 4D, respectively (**Supplementary Figure S3**). The regions might include candidate genes for *cqTN-2D.2* and *cqTN-4D.2*.

The closest markers of the seven stable cQTL were employed to detect effects in the RIL population (**Table 6**). The closest marker of *cqTN-2D.2* was *AX-109283238*, its biallelic polymorphic alleles were C//T, and the TN of homozygous alleles from CN18 were 250.88, 493.56, and 310.94 in the TNPW, MTN, and PTN growth stages, respectively. The TN of homozygous alleles from T1208 were 227.05, 382.06, and 267.36, respectively. The TN differences between the two genotypes of AA (from CN18) and aa (from T1208) were significant (*p* < 0.001).

**Table 6:** Effects of seven stable QTL in RIL population.

cQTL	Closest marker	Ordered alleles^a^	Stages	TN of AA^b^	TN of aa^c^	Difference (%)^d^	*p*-value
*cqTN-2D.1*	*AX-109945455*	G//A	MTN	492.17	378.60	23.08***	<0.001
			PTN	307.87	267.95	12.97***	<0.001
*cqTN-2D.2*	*AX-109283238*	C//T	TNES	97.77	93.28	4.59**	<0.01
			TNPW	250.88	227.05	9.50***	<0.001
			MTN	493.56	382.06	22.59***	<0.001
			PTN	310.94	267.36	14.02***	<0.001
*cqTN-4A*	*AX-108928080*	C//T	TNPW	234.03	238.54	1.89	>0.05
			MTN	415.34	438.10	5.20*	<0.05
			PTN	282.85	287.95	1.77	>0.05
*cqTN-4D.2*	*AX-110466464*	C//T	MTN	388.99	477.80	18.59***	<0.001
*cqTN-5A.1*	*AX-111496163*	A//G	TNPW	254.50	223.75	12.08***	<0.001
			PTN	303.48	271.68	10.48***	<0.001
*cqTN-5D.1*	*AX-110497303*	C//T	TNPW	249.69	229.22	8.20***	<0.001
			MTN	474.91	399.94	15.79***	<0.001
			PTN	303.59	274.81	9.48***	<0.001
*cqTN-7D*	*AX-94514350*	T//C	MTN	449.49	413.96	7.90***	<0.001


*^*a*^Ordered alleles represent the polymorphism type of the SNP marker of CN18 and T1208, respectively. ^*b,c*^AA represents homozygous alleles from CN18. aa represents homozygous alleles from T1208. Phenotypic values were measured for average of 2 years. ^*d*∗^, ^∗∗^, and ^∗∗∗^ indicate extremely significant correlation at 0.05, 0.01, and 0.001 probability levels, respectively.*

### Dynamic QTL Analysis for TN Across Different Growth Stages

**FIGURE 2:**
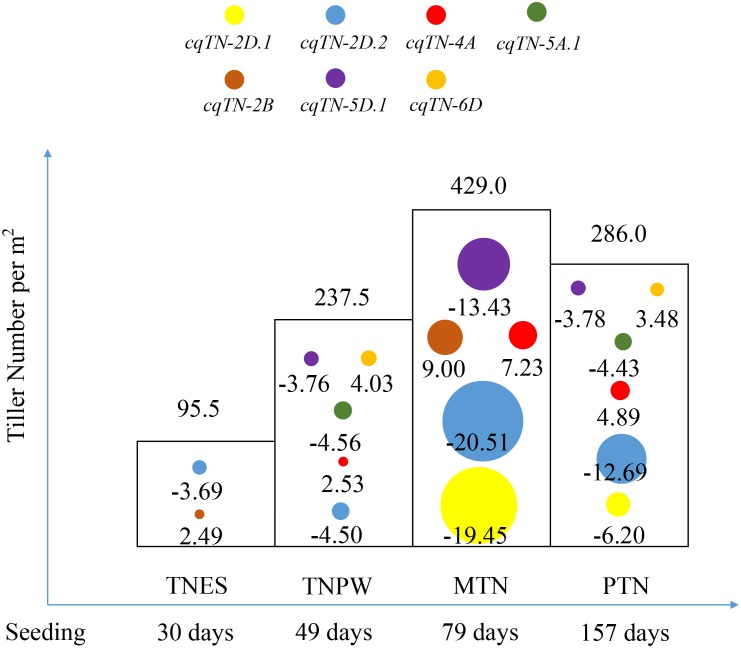
The dynamic QTL analysis for TN across four growth stages. Circles with different colors represent different QTL identified by combined QTL analysis. The value below each circle represents the additive effect of corresponding QTL. The value at the top of each column represents the average tiller number across the RIL population between 2 years. TNES, TNPW, MTN, and PTN represent the different growth stages of tillering.

The results of combined QTL analysis were used to perform dynamic QTL analysis, and a total of seven QTL were detected in different growth stages of tillering (**Table 4**, **Figure 2**, and **Supplementary Table S2**). These QTL were mapped to chromosomes 2B, 2D, 4A, 5A, 5D, and 6D, respectively. Among these QTL, *cqTN-2D.1*, *cqTN-2D.2*, *cqTN-4A*, *cqTN-5A.1*, and *cqTN-5D.1* were considered stable QTL and were co-segregated with the QTL identified in the individual environments. The *cqTN-2D.2* QTL was detected in the four growth stages, the LOD of *cqTN-2D.2* ranged from 4.47 to 55.28, and the LOD (A) and LOD (AE) ranged from 1.80 to 39.73 and 2.67 to 19.89, respectively. The total phenotypic variation explained (PVE) ranged from 4.92 to 17.06%, and the PVE (A) and PVE (AE) ranged from 2.43 to 13.25% and 2.49 to 4.40%, respectively (**Table 4**). Both *cqTN-5A.1* and *cqTN-6D* were detected at the TNPW and PTN growth stages with similar additive effects, but these two QTL did not express at the MTN growth stage (**Table 4** and **Figure 2**). The most important QTL cluster C1 was mapped to chromosome 2DS, where the beneficial alleles from CN18 increased TN at all growth stages (**Tables 5**, **6**).

## Discussion

### QTL Mapping for TN and Comparison With Other Studies

In the present study, seven stable QTL for TNES, TNPW, MTN, and PTN were mapped to different chromosomes (**Tables 3**, **4**). Among them, *cqTN-2D.1* at the MTN growth stage, *cqTN-2D.2* at the MTN and PTN growth stages, and *cqTN-5D.1* at the MTN growth stage were considered major. TN were affecting by the environmental factors, such as seeding density, sowing time and temperature, etc. In our previous study it was indicated that influence of weather was in a credible range during 2014–2016 (Tang et al., 2017). Moreover, the correlation of TNES, TNPW, MTN, and PTN between two experiment years were significant positive (*p* < 0.001) (**Table 1** and **Supplementary Figure S1**). These also could prove that the data were accurate and believable. The results of QTL and environment interaction analysis showed that the phenotypic variation explained by environment effects of *cqTN-2D.2* were small, indicated that *cqTN-2D.2* might be a stable QTL (**Table 4**). Huang et al. (2003) reported a QTL associated with TN mapped to chromosome 2DL. In addition, *Qltn.sicau-2D* was a major, stable QTL mapped to chromosome 2DL that reduces the tiller number in the H461/CN16 RIL population (Wang et al., 2016). However, *cqTN-2D.1* and *cqTN-2D.2* were both localized in a QTL cluster on chromosome 2DS, according to the results of BLASTN of the flanking SNPs against the Chinese Spring contigs. Xu et al. (2017) reported a major QTL for plant height and MTN on chromosome 2DS that is linked with SSR marker *Xcfd11*, which was located on the short arm far from the centromere based on the physical location of Chinese Spring from the United States Department of Agriculture^9^. Neither *cqTN-2D.1* nor *cqTN-2D.2* were the same as these QTL. Therefore, *cqTN-2D.1* and *cqTN-2D.2* were novel QTL for TN in wheat and have application value for MAS in breeding programs; the beneficial alleles of these QTL were both from CN18. We have reported a major QTL associated with TNPW and MTN, and this QTL (*cqTN-4D.1*) was also detected in the present study (**Supplementary Table S2**). As no polymorphism markers were detected between SSR markers *Xcfd23* and *Xwmc288*, we could not refine mapping of this QTL. However, it is conceivable that a QTL cluster mapped to chromosome 4D controls TN and related traits. Li et al. (2010) reported a QTL controlling TN mapped to chromosome 5D that is closely linked to SSR marker *Xwmc215*. The method of relative percentages was used by converting genetic distances to identify the relationship of this QTL and *cqTN-5D.1* (Wang et al., 2016). The results showed that the average relative percentage of *Xwmc215* was 57.48%, whereas the relative percentage of *cqTN-5D.1* in this study was 9.79%, indicating that these two QTL are not the same. A major QTL increasing TN was mapped to chromosome 5AL and linked to SSR marker *Xpsr370* (Kato et al., 2000), and it was proved that is near the end of chromosome 5AL associated with *Qltn.sicau-5A* (Wang et al., 2016). In the present study, *cqTN-5A.1* and *cqTN-5A.2* were also detected approximately 20 cM near the end of chromosome 5AL, and these QTL were considered to be linked or identical. In addition, Zou et al. (2017) reported a QTL cluster near the end of chromosome 5AL that was associated with flowering time and maturity in wheat using the 90K SNP Array. Based on various genetic backgrounds and different marker types, we assume that there is an enrichment region near the end of chromosome 5AL that controls several agronomic traits.

One hundred and thirty-six and fifty-six candidate genes of *cqTN-2D.2* (13.71 Mb) and *cqTN-4D.2* (7.36 Mb) were predicted by the flanking sequences of linkage SNP markers, respectively (**Supplementary Figure S3**). These candidate genes could provide more information of MTN and PTN. The results provide a better understanding of the QTL selectively expressing the control of tiller number and will facilitate future map-based cloning.

The BLASTX search was performed by using the flanking sequences of SNP markers as queries on ENSEMBL. 1,062 out of 11,583 (9.17%) SNP markers showed best hits to the Chinese Spring contigs (**Supplementary Table S3**). In the previously study, the Wheat660K Array hit 8.90% to the Chinese Spring contigs (Cui et al., 2017). It was indicated that Wheat55K Array was efficient and valid to construct a high-density wheat genetic map as Wheat660K Array.

### Dynamic Analysis of TN at QTL Level

The theory of developmental genetics suggests that QTL may have temporal expression during trait growth stages (Zhu, 1995; Liu et al., 2009). Therefore, it is necessary to identify the dynamic expression of QTL at different growth stages. The dynamic QTL analysis of rice TN and plant height has been reported (Liu et al., 2010, 2012), but dynamic QTL analyses of TN in wheat are limited. Xie et al. (2016) reported QTL cluster-associated tillering dynamics of wheat using RFLP and SSR markers that were mapped to chromosome 5AL. The dynamic analysis of wheat PH also showed a temporal expression pattern of QTL in various growth stages and environments (Wang et al., 2010). In the present study, a high-density genetic map derived from the Wheat55K SNP Array was employed for dynamic QTL analysis for TN and spike number (designated at PTN and considered as a growth stage of tillering) in wheat. *cqTN-2D.2* (**Table 4**) is a major, stable QTL located in QTL cluster C1 and mapped to chromosome 2DS, and the PVE of this QTL is <10% in the TNES and TNPW stages and >10% in MTN and PTN stages, which are the most important stages affecting final grain yield (Naruoka et al., 2011). In addition, the PVE (AE) of the QTL varied at 2.49–4.40%, indicating that this QTL was stable and less affected by environmental factors. Most QTL were detected at the TNPW, MTN, and PTN growth stages. More interestingly, two QTL exhibited dynamic changes across stages. *cqTN-5A.1* and *cqTN-6D* were detected at TNPW and PTN growth stages, with similar additive effects but were not expressed at MTN stage. These two QTL verified the theory of developmental genetics that temporal QTL may have similar final effects (Zhu, 1995; Liu et al., 2009). Both *cqTN-4D.2* and *cqTN-7D* were only expressed at the MTN stage; although previous studies demonstrated that MTN determines PTN (Li et al., 2010; Naruoka et al., 2011), there are QTL controlling tiller development but not tiller survival. Previous studies suggested that genes and QTL of high pleiotropy are expected to be under strong stabilizing selection (Hodgkin, 1998). Therefore, for breeding, *cqTN-2D.2* may be the best choice because it can be more stable across different environments. Furthermore, its additive effects are negative, indicating that beneficial alleles derived from CN18 increased TN at all growth stages. Understanding the temporal expression pattern of QTL based on dynamic analysis would help elucidate the genetic basis of tillering and help breeders decide when to utilize these QTL and their associated markers.

### Elite Alleles From CN18

Identification of useful alleles by association analysis has been an effective method for plant genomic research and MAS in recent years (Wang et al., 2016; Chen et al., 2017). In the present study, we verified the effects and beneficial alleles of QTL in the RIL population (**Table 6**). For instance, RILs with CN18 alleles at the *cqTN-2D.1*, on average, had 23.08% (*p* < 0.001) and 12.97% (*p* < 0.001) more TN than RILs with T1208 alleles, respectively. In addition, *cqTN-2D.2*, *cqTN-5A.1*, *cqTN-5D.1*, and *cqTN-7D* had the same effects in the RIL population. Only *cqTN-4A* had no significant differences in the RIL population, and the beneficial allele at *cqTN-4D.2* was from T1208. Almost all beneficial alleles were from CN18, and the lines that carry these elite alleles could provide valuable information in breeding programs. The validation of QTL effects in the RIL population is a powerful tool for identifying the molecular markers associated with quantitative traits in wheat.

Previous research has suggested that limited-tillering cultivars have higher yield-increasing potential than free-tillering cultivars in wheat and barley (Richards, 1988; Donald, 2009). However, this may not apply to Sichuan Province due to special environmental factors, such as fewer light hours and high humidity. CN18 is a multiple-spike wheat cultivar with high-tillering capacity, and the average spike formation rate of CN18 was 0.77 (Hu et al., 2017). According to the variety region trials in Sichuan Province, the yield of CN18 was 17.70% higher than the control cultivar (Ren et al., 2011), indicating that free-tillering cultivars had the same yield-increasing potential as limited-tillering cultivars in Sichuan Province and similar environments. For multiple-spike wheat cultivars, although some tillers die before harvest, small tillers contribute to the carbohydrate supply in spike formation (Lupton and Pinthus, 1969). Thus, beneficial alleles from CN18 at the QTL level are useful resources for elucidating the regulatory mechanisms underlying the genetic basis of tillering.

## Conclusion

In the present study, a high-density genetic map was constructed using the Wheat55K SNP Array for the first time in the CN18/T1208 RIL population; this map consisted of 371 lines. Seven stable QTL for TN at different growth stages were identified, and among these, *cqTN-2D.1*, *cqTN-2D.2*, and *cqTN-5D.1* were novel stable QTL. The beneficial alleles of these QTL were detected in the RIL population, and the beneficial alleles from CN18 at the QTL level and wheat lines with transgressive phenomena in the RIL population are useful germplasm resources. The present study improves our understanding of the genetic basis of TN and temporal expression of these QTL in wheat, as well as MAS and map-based cloning. The Wheat55K SNP Array is valid for wheat genetic mapping.

## Author Contributions

TR and ZL conceived the project. TR and ZR constructed the RIL population. TR, YH, YT, CL and ZL carried out the experiments. TR, YH, ZL, YT, ZR, FT, ZT, SF, and BY performed the phenotypic data. YH and TR analyzed the experimental results and wrote the manuscript.

## Conflict of Interest Statement

The authors declare that the research was conducted in the absence of any commercial or financial relationships that could be construed as a potential conflict of interest.

## Footnotes

1 https://www.ers.usda.gov/data-products/wheat-data.aspx

2 http://www.tsingke.net

3 http://www.affymetrix.com/support/downloads/manuals/axiom_analysis_suite_user_guide.pdf

4 http://www.isbreeding.net/

5 http://www.mybiosoftware.com/mapchart-2-2-graphical-presentation-linkage-maps-qtls.html

6 http://plants.ensembl.org

7 http://www.wheatgenome.org/

8 http://urgi.versailles.inra.fr/download/iwgsc/

9 http://wheat.pw.usda.gov/cgi-bin/cmap/

## Supplementary Material

**FIGURE S1 |** Phenotypic distribution of tiller number at early stage per unit area (TNES), tiller number during pre-winter per unit area (TNPW), maximum tiller number per unit area (MTN), and productive tiller number per unit area (PTN) in 2015 and 2016, respectively. The arrows indicate values of the parental lines: CN18 (C) and T1208 (T).

**FIGURE S2 |** Genetic map of CN18/T1208 RIL population constructed by Wheat55K SNP Array.

**FIGURE S3 |** Predicted genes of *cqTN-2D.2* (2D: 82189047-95895626) and *cqTN-4D.2* (4D: 374963016-382320415) in *Triticum aestivum*.

**TABLE S1 |** The detail information of each bin in linkage map construction.

**TABLE S2 |** QTL detected by multi-environmental analysis.

**TABLE S3 |** The coding region SNP (cSNP) marker mapped to the CN18/T1208 RIL population.
