# Supplementary figures and images for: Utilization of a Wheat55K SNP Array for Mapping of Major QTL for Temporal Expression of the Tiller Number
Source: Front Plant Sci. 2018 Mar 15;9:333. doi: 10.3389/fpls.2018.00333 (PMC5862827; doi:10.3389/fpls.2018.00333)

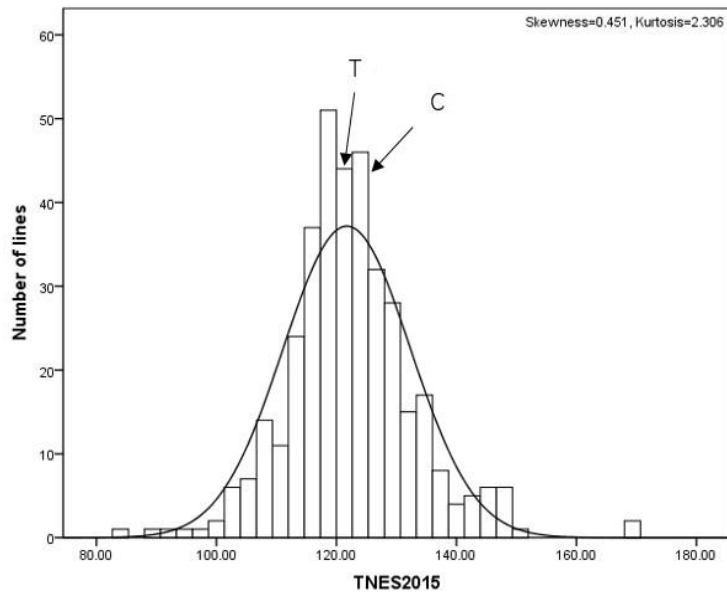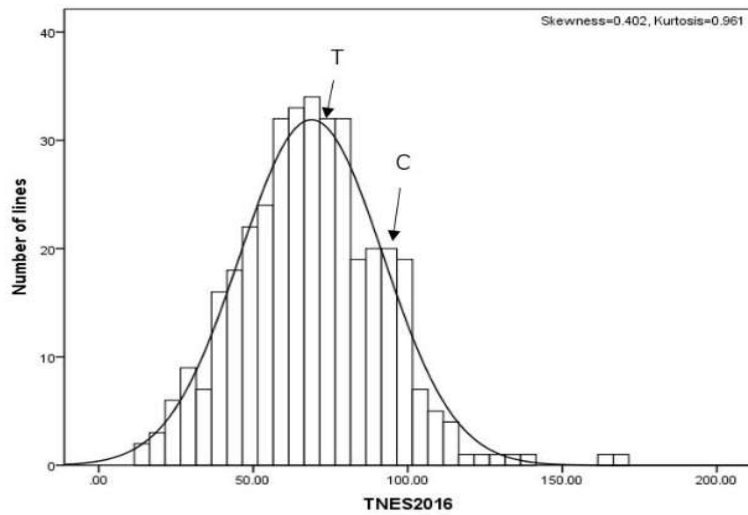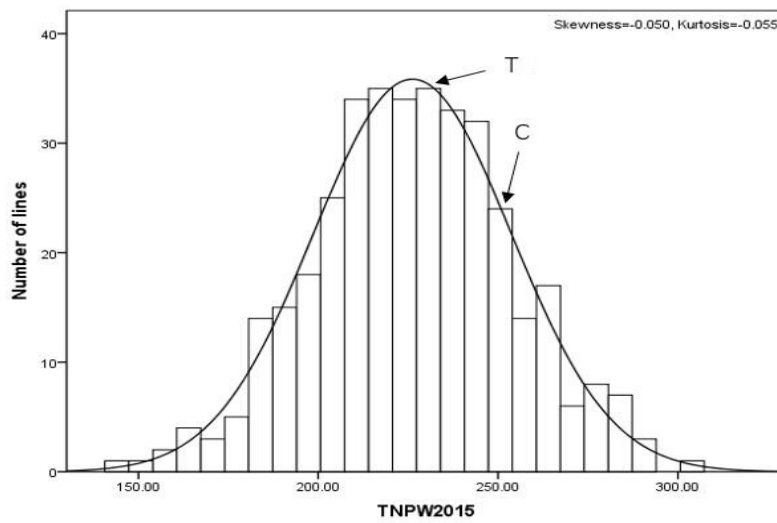

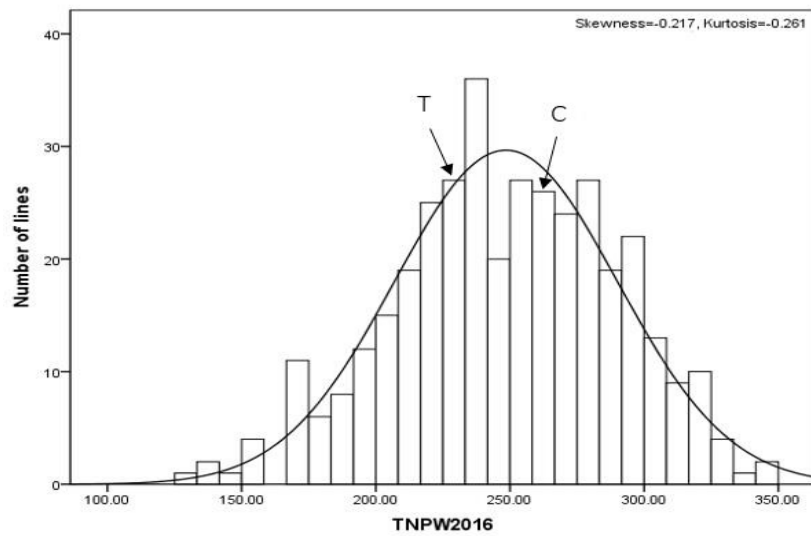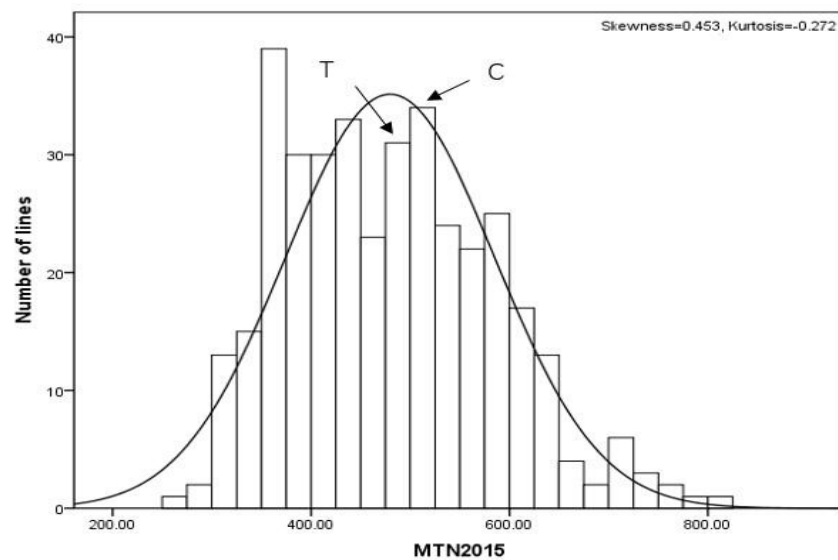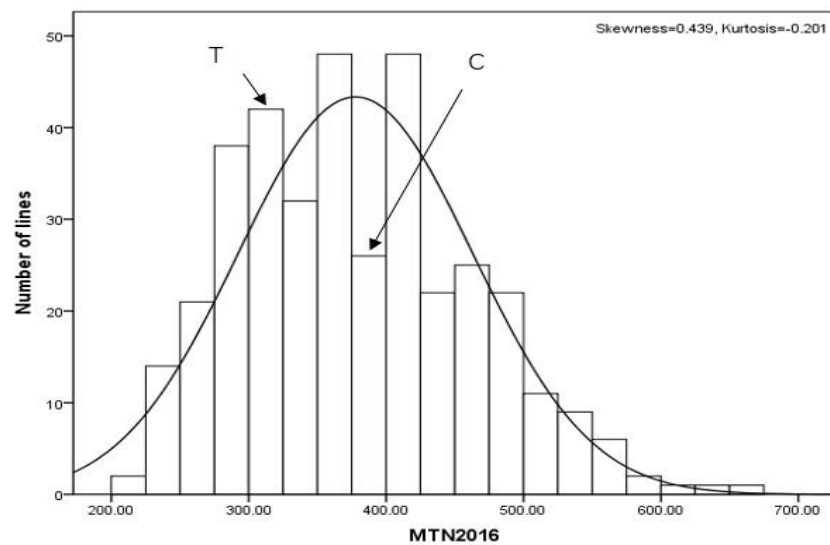

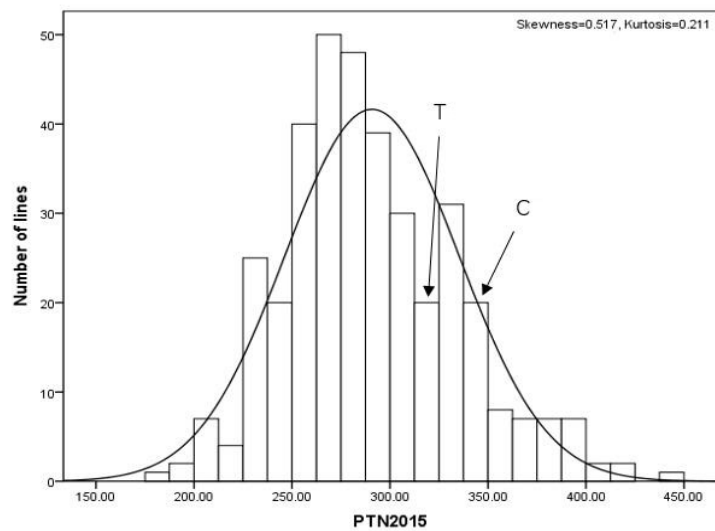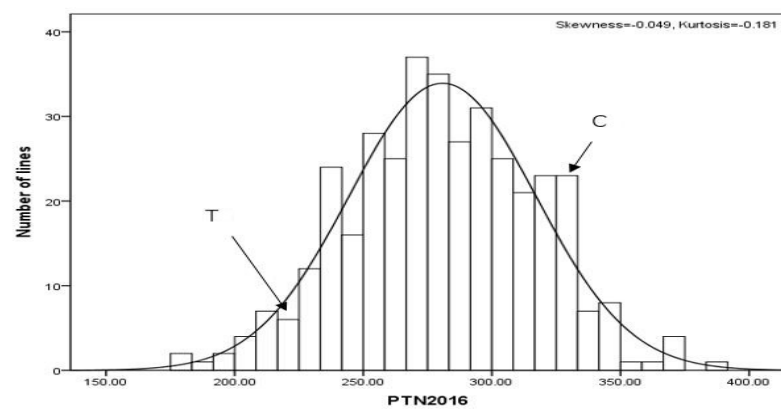

Supplement: Supplementary file 1 [file Image_1.PDF]

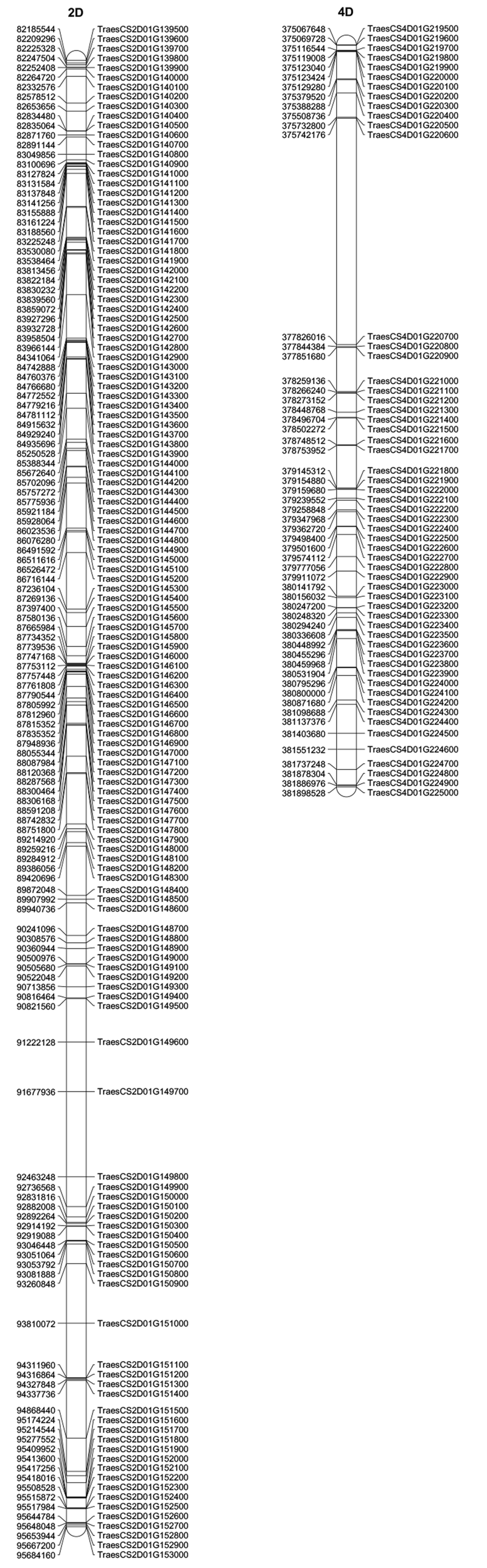

Supplement: Supplementary file 3 [file Image_3.JPEG]
