# Supplementary material for: Utilization of a Wheat55K SNP Array for Mapping of Major QTL for Temporal Expression of the Tiller Number
Source: Front Plant Sci. 2018 Mar 15;9:333. doi: 10.3389/fpls.2018.00333 (PMC5862827; doi:10.3389/fpls.2018.00333)
Supplement: Supplementary file 5 [file Table_2.DOCX]

Supplementary Table S2. QTL detected by muti-environmental analysis

| cQTL^a^ | Interval (cM) | Stage | LOD | LOD (A)^b^ | LOD (AE)^c^ | %PVE | %PVE (A)^d^ | %PVE (AE)^e^ | Add^f^ |
| --- | --- | --- | --- | --- | --- | --- | --- | --- | --- |
| *cqTN-1A* | 48.15-48.55 | TNES | 7.21 | 4.87 | 2.34 | 6.36 | 2.79 | 3.57 | 2.35 |
| *cqTN-2B* | 99.45-106.15 | TNES | 5.25 | 3.66 | 1.59 | 4.49 | 2.06 | 2.43 | 2.49 |
|  |  | MTN | 11.53 | 6.65 | 4.88 | 2.49 | 1.70 | 0.79 | 9.00 |
| *cqTN-2D.1* | 56.75-57.45 | MTN | 37.27 | 32.00 | 5.27 | 10.99 | 8.45 | 2.54 | -19.45 |
|  |  | PTN | 22.60 | 9.66 | 12.94 | 6.01 | 3.23 | 2.78 | -6.20 |
| *cqTN-2D.2* | 70.45-81.75 | TNES | 4.47 | 1.80 | 2.67 | 4.92 | 2.43 | 2.49 | -3.69 |
|  |  | TNPW | 8.19 | 4.82 | 3.37 | 6.90 | 3.06 | 3.84 | -4.50 |
|  |  | MTN | 55.28 | 35.39 | 19.89 | 13.62 | 9.22 | 4.40 | -20.51 |
|  |  | PTN | 47.05 | 39.73 | 7.32 | 17.16 | 13.25 | 3.91 | -12.69 |
| *cqTN-4A* | 122.75-137.05 | TNPW | 5.34 | 1.55 | 3.79 | 1.73 | 1.00 | 0.73 | 2.53 |
|  |  | MTN | 11.42 | 4.68 | 6.74 | 2.88 | 1.20 | 1.68 | 7.23 |
|  |  | PTN | 7.48 | 6.13 | 1.35 | 2.64 | 2.00 | 0.64 | 4.89 |
| *cqTN-4D.1* | 38.35-41.55 | TNPW | 8.90 | 1.46 | 7.44 | 4.03 | 0.87 | 3.16 | 2.44 |
| *cqTN-4D.2* | 72.35-78.35 | MTN | 25.67 | 11.86 | 13.81 | 6.48 | 3.07 | 3.41 | 11.59 |
| *cqTN-5A.1* | 141.35-151.95 | TNPW | 10.64 | 5.07 | 5.57 | 3.70 | 3.19 | 0.51 | -4.56 |
|  |  | PTN | 9.00 | 5.30 | 3.70 | 2.36 | 1.65 | 0.71 | -4.43 |
| *cqTN-5A.2* | 166.95-171.55 | TNES | 23.69 | 8.10 | 15.59 | 6.09 | 4.53 | 1.56 | -2.91 |
| *cqTN-5D.1* | 9.85-25.35 | TNPW | 5.59 | 2.66 | 2.93 | 3.55 | 1.72 | 1.83 | -3.76 |
|  |  | MTN | 69.52 | 14.82 | 54.70 | 21.22 | 3.87 | 17.35 | -13.43 |
|  |  | PTN | 7.26 | 3.17 | 4.09 | 1.63 | 0.99 | 0.64 | -3.78 |
| *cqTN-5D.2* | 210.15-212.05 | MTN | 9.35 | 3.69 | 5.66 | 2.17 | 0.95 | 1.22 | -6.71 |
| *cqTN-6D* | 271.06-279.36 | TNPW | 4.58 | 2.50 | 2.08 | 2.43 | 1.60 | 0.83 | 3.48 |
|  |  | PTN | 4.48 | 3.62 | 0.86 | 1.78 | 1.17 | 0.61 | 4.03 |
| *cqTN-7D* | 193.95-199.25 | MTN | 10.02 | 7.12 | 2.90 | 2.33 | 1.70 | 0.63 | -8.98 |

^a^ cQTL represent QTL identified by the combined QTL analysis of multi-environment trials. These cQTL in ***bold*** typeface were the stable QTL and showed in Table 4 of the manuscript. These cQTL in *underline* typeface were used to the dynamic analysis of TN.

^b, c^ LOD (A) represent LOD score for additive and dominance effects. LOD (AE) represent LOD score for additive and dominance by environment effects.

^d, e^ %PVE (A) and %PVE (AE) represents phenotypic variation explained by additive and dominance effect and by additive and dominance by environment effect for the corresponding QTL, respectively.
